# Reviewers for *Journal of Insect Science* (November 2021–October 2022)

**DOI:** 10.1093/jisesa/iead001

**Published:** 2023-06-03

The Editor-in-Chief and the Subject Editors of *Journal of Insect Science* thank the following scientists for their voluntary commitment of valuable professional time and expertise to peer reviewing manuscripts submitted for publication in our journal. The quality and scientific stature of the journal depends on the conscientious efforts of these individuals.

## Top reviewers (3+ reviews completed)

Cai, Yao

Klinger, Ellen

## Reviewers

Ab Majid, Abdul Hafiz

Adams, Richard

Alananbeh, Kholoud M.

Alburaki, Mohamed

Aluja, Martin

Amsalem, Etya

Anbalagan, Sankarappan

Angelella, Gina

Athanassiou, Christos

Atlihan, Remzi

Audisio, Marcela Carina

Avtzis, Dimitrios

Ayvaz, Abdurrahman

Azmi, Wahizatul Afzan

Bakhsh, Allah

Barbero, Francesca

Barreto, Carlos

Baur, Bruno

Beer, Chantel de

Bendena, William

Bonnard, Michel

Borges, Paulo A. V.

Boulton, Rebecca

Bovera, Fulvia

Bruninga-Socolar, Bethanne

Capriotti, Natalia

Carroll, Mark

Catalano, María Inés

Chaisuekul, Chatchawan

Chaudhury, Muhammad

Chawla, Geetanjali

Chen, Mingshun

Chen, Yolanda

Ciampor, Fedor

Coop, Leonard

Corronca, José

Cortés, Nancy

Daisley, Brendan

Danihlik, Jiri

Davis, Jeffrey

Del-Claro, Kleber

Dharavath, Neethu

Di, Xueyuan

do Nascimento, Antonio

Dominiak, Bernie

Durant, Jennie

Emlen, Douglas

Faiman, Roy

Falabella, Patrizia

Faltýnek Fric, Zdeněk

Fathipour, Yaghoub

Fauvergue, Xavier

Fereres, Alberto

Ferrante, Marco

Fine, Julia

Flores, Salvador

Floros, George

Flynn, Peter

Foster, Leonard

Freelance, Christopher

Fronza, Georgina

Furlan, Lorenzo

Geib, Scott

Genta, Fernando

Gerken, Alison

Gourgouta, Marina

Graf, Joerg

Groß, Julia

Guo, Dezheng

Gurung, Kiran

Hagler, James

Harmon, Jason

Harpur, Brock

Hatakeyama, Masatsugu

He, Ming

He, Peng

Heckel, David

Hee, Alvin Kah Wei

Hepler, James

Hernandez, Emilio

Hingston, Andrew

Hinkelman, Travis M.

Hoffmann, Ary

Homberg, Uwe

Huang, Qiuying

Hubert, Jan

Hui, Jerome H. L.

Hulse-Kemp, Amanda

Hunter, David

Huston, Daniel

Hwang, Shaw Yhi

Inouye, David W.

Jack, Cameron

Janowiecki, Mark

Jaronski, Stefan

Jaumann, Sarah

Johnson, Reed

Jung, Jin Kyo

Jurenka, Russell

Kajtoch, Łukasz

Keena, Melody

King, Bethia

Kittelmann, Sebastian

Kojima, Wataru

Ksentini, Ines

Kus, Krzysztof

Lara, Ricky

Lavore, Andres

Lee, Benjamin

Lee, Jana

Li, Bin

Li, Hu

Li, Junlan

Li, Zhilin

Liao, Ling-Hsiu

Li-Byarlay, Hongmei

Liebhold, Andrew

Liu, Cunchen

Liu, Wen

Liu, Xusheng

Liu, Yan-Qun

Lobo, Jorge

Lourenco, Anete

Lu, Yanhui

Ludwick, Dalton

Ma, Sanyuan

Ma, Weihua

Maekawa, Kiyoto

Mahdian, Kamran

Malysh, Julia M.

Managanvi, Kamlesh

Mastrantonio, Valentina

Metz, Bradley

Millar, Jocelyn G.

Minaei, Kambiz

Mizumoto, Nobuaki

Mo, Jianchu

Mohamed, Amr

Mola, John

Molleman, Freerk

Montoya, Pablo

Morrison, William

Nègre, Nicolas

Nixon, Laura

Nunes, Francis

Ons, Sheila

Ovruski, Sergio

Packard, Gary C.

Pallipparambil, Godshen

Pang, Hong

Paredes-Montero, Jorge R.

Park, Yoonseong

Pehlivan, Serkan

Poelchau, Monica

Pomeroy, Nelson

Popadic, Aleksandar

Popham, Holly

Prade, Patricia

Prasifka, Jarrad

Prosser, R. S.

Quesada-Moraga, Enrique

Ramirez, Claudio C.

Rault, Leslie

Reinders, Jordan

Reitz, Stuart

Ren, Bingzhong

Rhode, Clint

Rodovalho, Cynara

Roggero, Angela

Rohlfs, Marko

Romanholo Ferreira, Luiz Fernando

Rossini, Michele

Roulston, T’ai

Rull, Juan

Rumbos, Christos

Sahragard, Ahad

Sakurai, Takeshi

Scharf, Inon

Scherer, Brendan

Scieuzo, Carmen

Scott, Ian

Scott, Maxwell

Seal, Dakshina

Serbina, Lillya

Severtson, Dustin

Sharma, Anamika

Shegelski, Victor

Shi, Fu-Ming

Simo, Ladislav

Sless, Trevor

Smith, Adam

Smith, Hugh

Song, Nan

Sreenivasamurthy, Sreelakshmi

St. Clair, Coy

Starkie, M. L.

Stavenga, D. G.

Su, Jianya

Su, Lijuan

Sun, Ruo

Sun, Shou-Hui

Suraporn, Siripuk

Swale, Daniel

Tait, Cheyenne

Tammaru, Toomas

Tang, Bin

Teale, Stephen

Terra, Walter

Thangavel, Boopathi

Thomaes, Arno

Toledo, Jorge

Tonğa, Adil

Toth, Amy

Trematerra, Pasquale

Urban, Julie

Valles, Steven M.

Van Eeckhoven, Jens

van Etten, Megan

Vavassori, Laura

Venthur, Herbert

Vyavhare, Suhas

Wagner, Anika E.

Wan, Hu

Wang, Xiao-yi

Wang, Xingeng

Waterhouse, Robert

Wei, Zhao-Jun

Wiemers, Martin

Wiman, Nik

Woller, Derek

Woodard, Hollis

Wu, Jianing

Wu, Yunke

Xiao, Da

Xu, Haijun

Xu, Wei

Yan, Fengming

Yang, Chin-Cheng Scotty

Yang, Maofa

Yang, Xueqing

Yuan, Jiazheng (John)

Zalucki, Myron

Zaviezo, Tania

Zhang, Feiping

Zhang, Li

Zhang, Long-Wa

Zhang, Qi-Lin

Zhang, Qing-he

Zhang, Wei

Zhang, Zhen

Zhang, Zhengqun

Zhou, Wen-Wu

Zhu, Lieceng

Zong, Shixiang

Zuniga, Gerardo

